# Real-time powder diffraction studies of energy materials under non-equilibrium conditions

**DOI:** 10.1107/S2052252517010363

**Published:** 2017-09-01

**Authors:** Vanessa K. Peterson, Josie E. Auckett, Wei-Kong Pang

**Affiliations:** a Australian Nuclear Science and Technology Organisation, Locked Bag 2001, Kirrawee DC, NSW 2232, Australia; b Institute for Superconducting and Electronic Materials, Faculty of Engineering, University of Wollongong, Wollongong, NSW 2522, Australia

**Keywords:** real-time studies, *operando* studies, powder diffraction, functional materials, energy materials

## Abstract

Energy materials form the central part of energy devices. An essential part of their function is the ability to reversibly host charge or energy carriers, and analysis of their phase composition and structure in real time under non-equilibrium conditions is mandatory for a full understanding of their atomic-scale functional mechanism. Real-time powder diffraction is increasingly being applied for this purpose, forming a critical step in the strategic chemical engineering of materials with improved behaviour. This topical review gives examples of real-time analysis using powder diffraction of rechargeable battery electrodes and porous sorbent materials used for the separation and storage of energy-relevant gases to demonstrate advances in the insights which can be gained into their atomic-scale function.

## Abstract 2

This topical review presents developments in the analysis of functional energy materials using real-time *operando* X-ray and neutron powder diffraction of non-equilibrium systems. Examples in the areas of porous framework materials and rechargeable battery electrodes are used to highlight improvements in structural detail gained for guest–host systems through advances in instrumentation and experimental approach.

## 1. Introduction   

Functional energy materials form the central part of many important technologies used for the storage, transport and delivery of energy. Their atomic structure influences their chemical and physical properties, which in turn underpin the performance characteristics of energy devices. These materials must often reversibly host charge or energy carriers, and characterizations focused on understanding how these guests are accommodated by the host material are central to technological advancement. How the charge or energy carrier (molecule) enters, moves through and is stored within the host, as well as the corresponding reverse processes that release the charge or energy carrier, all need to be understood.

Time-resolved powder diffraction is an established tool for the investigation of processes and kinetics (Eckold *et al.*, 2010 ▸; Evans & Evans, 2004 ▸; Leineweber & Mittemeijer, 2012 ▸; Norby, 1996 ▸). Whilst studies of non-equilibrium systems using powder diffraction are commonplace, and include crystallization (Simmance *et al.*, 2015 ▸) and other reactions (Hansen & Kohlmann, 2014 ▸), the use of the method to study guest–host systems is less common. *In situ* diffraction experiments under variable conditions of guest concentration, temperature, charge rate *etc*. are widely conducted on such systems, but the vast majority of these involve measurements of the system only at equilibrium at discrete points in the parameter space, with the assumption that the system’s response to the external stimulus is correctly represented. Such *in situ* studies of the system at equilibrium are increasingly being recognized as not representative of the structural processes occurring during real-time function, and there are examples, notably in battery electrodes, where this assumption is demonstrably incorrect (Grey & Tarascon, 2017 ▸).

Ideally, studies of functional host–guest systems should probe guest transport mechanisms during function within a device or during the material’s response to external stimuli (*operando* studies), to avoid obtaining misleading or incomplete results from the equilibrium system. Because all powder diffraction data represent a time-averaged measurement, the distribution of states that are represented in the sample during the period of data acquisition must be taken into account, complicating the structural analysis. *Operando* diffraction experiments are therefore relatively demanding, as they often require the sample of interest to be probed within a device and with sufficiently fast acquisition times to represent the process being measured. The level of detail gained about the material structure is in direct competition with the level of detail gained concerning its evolution. Several strategies can be taken to keep the distribution of states small in any particular measurement, including:

(i) Reducing the rate of change of the material response, such as by reducing the system temperature in order to influence the reaction kinetics.

(ii) Tuning instrumental optics in order to trade some angular resolution for increased flux (resolution/intensity), thereby decreasing data acquisition time.

Functional materials analysis is greatly enhanced by modern high-speed instrumentation, which allows the rapid collection of high-quality data and real-time information concerning the functioning/responding material. In-house laboratory X-ray diffractometers can be customized and dedicated to a particular type of experiment, offering an advantage over diffractometers at large-scale synchrotron and neutron radiation facilities which are shared internationally and tend to be multipurpose. Conversely, advances in sources and instrumentation at many synchrotron and neutron scattering centres, including large and sensitive detectors, are increasingly facilitating cutting-edge *operando* powder diffraction experiments. The higher flux available at synchrotron sources translates directly into higher spatial and temporal resolution powder diffraction than most neutron and laboratory X-ray sources can achieve. On the other hand, the nucleus–neutron interaction in neutron powder diffraction allows non-destructive measurement of the bulk, in combination with isotopic information and superior sensitivity to very light elements such as hydrogen and lithium in the presence of heavier elements. The lack of reduction in neutron scattering intensity with increased scattering angle also allows relatively more fine structural information to be gained than when using X-rays.

In the present topical review, we highlight recent developments in the analysis of functional energy materials using X-ray and neutron powder diffraction real-time *operando* measurements of non-equilibrium systems. Examples are chosen to highlight the increased level of structural detail gained for guest–host systems through advances in instrumentation and experimental approach, and are drawn from the literature and from our own experience investigating guest–host systems that relate to energy, most notably porous framework materials and rechargeable battery electrodes.

## 2. Porous framework materials   

Porous framework materials, such as metal–organic frameworks (MOFs), are a large class of materials with unprecedented structural and chemical diversity. They are composed of a regular arrangement of ‘nodes’, consisting of single atoms or ions (usually metals) or a cluster of these, that are coordinated to ligand molecules (usually organic) to form porous structures. Their relative ease of synthetic modification allows the chemical engineering of materials with targeted pore shapes, sizes and chemical functionality.

Porous frameworks can separate and reversibly store guest molecules such as hydrogen, methane and carbon dioxide from a range of industrially relevant mixtures, making them potentially useful as solid sorbents for energy systems. Compact hydrogen storage (Morris & Wheatley, 2008 ▸), post-combustion separation and capture of carbon dioxide from power plant flue gas or from ‘sour’ natural gas (Li *et al.*, 2011 ▸; Bae & Snurr, 2011 ▸), and cryogen-free purification of component gases from air (Li *et al.*, 2009 ▸) are among the many intended applications for solid sorbents that are currently under pursuit. A number of the functional properties of sorbent materials must be optimized in order to achieve these targeted applications, including the total adsorption capacity, degree of selectivity for the target species, kinetic efficiency of guest uptake, robustness to operating conditions (temperature and chemical environment) under repeated cycling and the strength of guest binding, which determines the energy cost of sorbent regeneration.

Guest–host properties in porous frameworks are commonly determined by coupling structural studies of these crystalline materials with simultaneous gas- or vapour-adsorption measurements. *In situ* studies of guest-adsorption properties have revealed important structure–function relationships in a large number of framework materials to date (Carrington *et al.*, 2014 ▸, and references therein), enabling progress towards targeted design. Both X-ray and neutron *in situ* powder diffraction measurements of the guest–host system are increasingly being performed for multiple guest types and over variable guest concentrations within the same host sample. This approach to studying the equilibrium system *in situ* can yield information about the location and preference order of binding sites, the relative uptake of different guests (an indicator of selectivity) and the average framework response to the presence of guests under various conditions of concentration and temperature. A typical example of such an approach is the series of *in situ* synchrotron powder diffraction measurements of gas adsorption in the porous framework material Cu_2_(pzdc)_2_(pyz) (pzdc = pyrazine-2,3-di­carboxylate and pyz = pyrazine) carried out on the BL02B2 beamline at the SPring-8 facility in Japan (Kitaura *et al.*, 2002 ▸; Matsuda *et al.*, 2005 ▸; Kubota *et al.*, 2005 ▸, 2006 ▸, 2007 ▸). The material was exposed to a constant pressure of O_2_, H_2_ or C_2_H_2_ and powder diffraction data were collected after equilibration of the system at various temperatures, with the chosen temperature modulating the guest concentration. Although measuring the equilibrium system, these *in situ* measurements as a function of guest concentration revealed a metastable adsorption state in the guest adsorption process (Kubota *et al.*, 2006 ▸). Pioneering experiments such as these paved the way for later powder diffraction measurements of porous framework–guest systems under non-equilibrium conditions, which are of particular importance for elucidating the entire mechanism of material function, such as reaction pathways in host-catalysed guest–guest reactions.

Until recently, progress towards achieving *operando* structural characterization during gas sorption processes in porous materials has been hampered by technical challenges associated with maintaining dynamic control of temperature (from the cryogenic range to ‘realistic’ operating conditions ≤ 350 K) and gas pressure (vacuum to ∼10^2^ bar; 1 bar = 100 000 Pa) while simultaneously collecting diffraction data from the unobstructed sample. This is somewhat easier to achieve at neutron sources than at X-ray sources, due to the superior beam penetration of neutrons into complex sample environments, as well as the larger sample sizes that are typically used to compensate for the order of magnitude lower scattering of neutrons from matter relative to X-rays. Larger sample sizes also allow gas dosing to be performed with greater quantitative accuracy. Nevertheless, studies recently conducted at several high-flux synchrotron X-ray sources have successfully utilized continuous-flow gas delivery systems suitable for *in situ* or *operando* X-ray scattering experiments on porous materials (He *et al.*, 2016 ▸; Salas-Colera *et al.*, 2015 ▸; Stoeckel *et al.*, 2015 ▸). Fast high-intensity neutron diffractometers that are able to acquire high-quality diffraction data containing meaningful structural information on a time scale of minutes or less, such as the General Materials Diffractometer (GEM) at the ISIS facility in the UK, the D20 instrument at the ILL facility in France, and WOMBAT at the Open-Pool Australian Lightwater (OPAL) research reactor, Australia, are also particularly well suited for the development of real-time gas delivery capabilities (Chevreau *et al.*, 2015 ▸).

Despite these advances, a recent search of the literature revealed a dearth of published characterization studies of porous materials combining *operando* gas sorption with diffraction of the non-equilibrium system, indicating that wide-scale adoption of such methods for investigating functional porous materials is yet to be realised.

### 2.1. First time-resolved measurements of a non-equilibrium framework–guest system   

**Figure 1:**
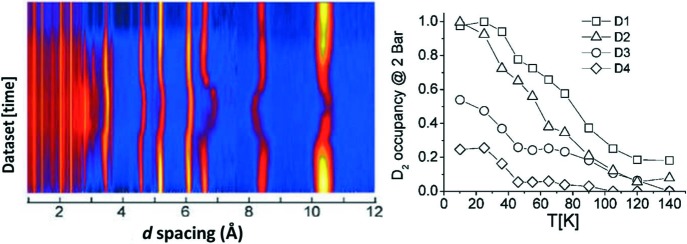
Neutron powder diffraction data for Cr(OH)(1,4-benzenedicarboxylate) loaded with D_2_, collected following initial loading at 25 K (measuring at 10 K) and during subsequent heating to 150 K under a constant pressure of 2.0 bar D_2_. The plot shows the site occupancies obtained from Rietveld refinement for D_2_ at four locations in the framework. Reprinted and adapted with permission from Mulder *et al.* (2010 ▸). Copyright (2010) American Chemical Society.

The first powder diffraction measurements of a non-equilibrium gas-porous framework system were reported for molecular hydrogen (as deuterium, D_2_) in Cr(OH)(1,4-benzenedicarboxylate) (Mulder *et al.*, 2010 ▸). Neutron powder diffraction data were collected on GEM at the ISIS facility in the UK. Data were recorded over 10–15 min during adsorption and desorption under 2 bar of D_2_ on heating (Fig. 1 ▸). The lattice parameter of the host framework was easily obtained from these data. Furthermore, difference Fourier methods allowed the identification of four binding sites arising from D_2_, called D1, D2, D3 and D4, and their depopulation under 2 bar of D_2_ during heating was found to occur in the order weakest to strongest.

### 2.2. Sub-minute time resolution: the time-resolved diffraction structure envelope approach   

**Figure 2:**
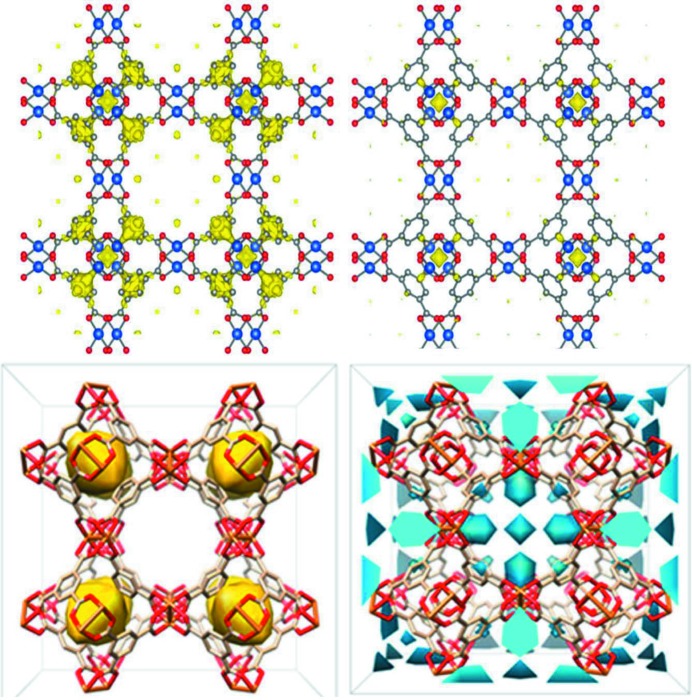
The nuclear density (yellow) arising from the 1.3 CD_4_ molecules per Cu (top row) in Cu_3_(btc)_2_ and the electron density (in yellow and blue) attributed to 1 bar of CH_4_ in Cu_3_(btc)_2_ obtained using TRDSE methods (bottom row) at 150 K (left) and 295 K (right). The densities are shown superimposed on one unit cell of Cu_3_(btc)_2_ viewed along [1, 0, 0]. Cu_3_(btc)_2_ is shown with Cu in blue (top) and orange (bottom), C in grey and O in red, and with H atoms omitted for clarity in all panels. Reprinted with permission from (top) Hulvey *et al.* (2015 ▸) and (bottom) Chen *et al.* (2015 ▸). Copyright (2015) American Chemical Society.

The time-resolution of the measurement time for guest–host structures during gas adsorption, desorption and equilibration using synchrotron powder X-ray diffraction was improved (Chen *et al.*, 2015 ▸) using the differential electron density (DED) approach (Yakovenko *et al.*, 2014 ▸). In the DED approach, a structure envelope of electron density is calculated *via* a Fourier transform using only those reflections in the powder diffraction pattern that change significantly upon guest loading, simplifying the data analysis. The DED is calculated by the difference between the measured structure envelopes for a sample loaded with guests and that expected for the material without guests. The DED is therefore a type of low-resolution difference electron-density map from which structural information about the host and/or pore guests can be derived. This simplified analysis was extended to the real-time analysis of guest dynamics in a framework host by introducing the time-resolved diffraction structure envelope (TRDSE) (Chen *et al.*, 2015 ▸). In this work, sequential synchrotron X-ray powder diffraction data sets were acquired for Cu_3_(btc)_2_ (btc = 1,3,5-benzenetricarboxylate) and three other porous coordination frameworks under 1 bar of CO_2_ and CH_4_. Data were recorded every 30 s on the 17-BM beamline of the Advanced Photon Source (APS), Argonne National Laboratory, USA, and repeated for three different temperatures. The results show that gas molecules generally prefer to redistribute over heterogeneous types of sites rather than exclusively occupy primary binding sites. Notably, these results are significantly different from those obtained using neutron powder diffraction to investigate the mechanism of deuterated methane (CD_4_) incorporation into Cu_3_(btc)_2_ by studying the material at equilibrium with particular guest concentrations (Hulvey *et al.*, 2015 ▸, Fig. 2 ▸). In the latter work, difference Fourier nuclear-density maps for the CD_4_-loaded Cu_3_(btc)_2_ equilibrium system show the localization of CD_4_, and Rietveld refinements revealed a small amount of CD_4_ inside the octahedral cages and at the window sites, with CD_4_ mostly absent from the coordinatively unsaturated Cu.

Hulvey *et al.* (2015 ▸) note these differences and discredit time-resolved work investigating guest sorption in porous frameworks, claiming that powder diffraction cannot be applied to systems not in equilibrium due to the potential for variability in the number of crystallographic phases present at any given time during the measurement of a constantly changing material. In reality, powder diffraction data contain information limited to the powder-averaged structure, and whilst not requiring every powder grain to be in the same state, a consideration of the various domains and phases in the system is required. The time-averaging of a dynamic system may be treated analogously. Data analysis must take into account that parts of a sample may not be in the same state at all times, and that attributing all features to a single phase may not be correct. For gas-loaded porous frameworks, the possibility that local guest concentrations may vary over time must be considered, just as the possibility that local concentrations of guests may (and often do) vary across the sample must also be considered.

The study of the guest–host equilibrium system clearly shows preferential sites for interaction of the methane with the Cu_3_(btc)_2_ host, and how the host responds to that (Hulvey *et al.*, 2015 ▸), but do not directly show the adsorption process. Conversely, the time-resolved work assumes that the guest–host system and the empty host at that temperature are the same, neglecting the substantial structural changes that occur in the host system on guest absorption (Chen *et al.*, 2015 ▸). The TRDSE captures only a few low-angle reflections, and along with the electron-density measurement brings elemental contrast issues typical of these host–guest systems, introducing further inaccuracy. The results of the time-resolved study (Chen *et al.*, 2015 ▸) may therefore not accurately reveal the mechanism for methane adsorption by Cu_3_(btc)_2_, but not because of the non-equilibrium approach. The assumption made in the time-resolved study of the non-equilibrium system (Chen *et al.*, 2015 ▸) is that the difference in peak intensity arises solely from the scattering of gas molecules and that the DED will therefore constitute the distribution of these in the unit cell, and the authors correctly note that the method is only valid when there is no phase transition and a negligible ‘breathing’ effect in the framework in response to gas adsorption.

### 2.3. Minute-scale time resolution: full Rietveld analysis   

Methane-rich natural gas hydrates are framework materials comprised of water molecule cages that form in the presence of gas and water within well defined pressure–temperature zones. Naturally occurring oceanic gas hydrates possess an estimated global carbon content at least as large as the total inventory of coal, oil and conventional gas sources. Gas hydrates are considered an alternative source of energy and intense scientific efforts have advanced such proposals to the stage of field trials. Existing exploitation ideas are currently focused on the destruction of the hydrate through de­pressurization, warming or the exchange of guests. Real-time powder diffraction of the non-equilibrium system, in particular using neutrons, has facilitated important advances in understanding these ideas. The determination of a representative composition requires a measurement over larger sample volumes for which powder diffraction is well suited. This has been successfully demonstrated on numerous occasions in the case of hydrate formation and decomposition (Falenty *et al.*, 2013 ▸; Falenty & Kuhs, 2009 ▸) and gas replacement involving structural changes (Murshed *et al.*, 2010 ▸; Halpern *et al.*, 2001 ▸). The decomposition is understood in terms of heat transfer and permeability of the host, but until recently there was no quantitative description of the exchange process, with a major obstacle being a lack of information on the time-resolved composition of the two-phase fluid at the gas–hydrate interface. This arises because the highly locally heterogeneous exchange reaction complicates the analysis.

**Figure 3:**
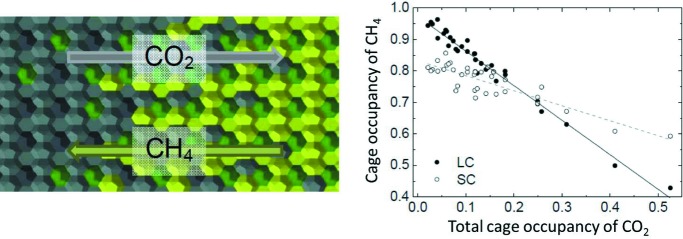
(Left) Schematic of gas exchange in a hydrate, proceeding through transport of CO_2_ from the particle surface to the core and the opposite migration of CH_4_. (Right) CH_4_ cage occupancies in large and small cages (LC and SC, respectively) as a function of CO_2_ content. Reprinted and adapted with permission from Falenty *et al.* (2016). Copyright (2016) American Chemical Society.

Falenty *et al.* (2016 ▸) overcame this issue by using a combination of space- and time-resolved information obtained by cryo-scanning electron microscopy, Raman spectroscopy and neutron powder diffraction measurements. Neutron powder diffraction measurements targeting CH_4_–CO_2_ exchange reactions in gas hydrates were performed on the high-flux two-axis neutron diffractometer D20 at the Institut Laue–Langevin (ILL) in France, where instrumental optics were optimized for fast data acquisition at the expense of some angular resolution (Falenty *et al.*, 2016 ▸). Temperature control was provided by a controller attached to an He-flow cryostat and samples were loaded in a pressure cell under pure CH_4_ gas pressure. Fluid in the pore spaces was exchanged by rapid pressure release and recompression with CO_2_, during which data were collected over a minimum of 60 s. The data were of sufficient quality to overcome difficulties in previous approaches that were only able to follow the cage occupancies by the evolution of the intensity ratio of ‘guest-sensitive’ reflections (Hansen *et al.*, 2016 ▸). Full Rietveld analysis for every acquisition allowed the simultaneous refinement of the mixed-hydrate weighted fractions and the lattice constants. Whilst the occupancies of CO_2_ and CH_4_ in large cages could be determined simultaneously, this could not be achieved in small cages due to the partial cancellation of the scattering signal from both guest gases and the low concentration of CO_2_ in a small cage. Consequently, for one data set (Fig. 3 ▸) the occupancy of guests in small cages was fixed at 5%, guided by Raman spectroscopic analysis of the recovered samples.

### 2.4. Fast time-resolved diffraction with accurately quantified guest uptake   

In the examples described above, the concentration of guest molecules present in the host framework during the experiment was neither controlled nor measured accurately. In cases where progressive guest loading triggers significant structural or behavioural change in the framework, determination of the threshold guest concentration required to induce the change yields information which may be critical to obtaining full understanding of the causal mechanism. Limitations on the design of sample environments for *operando* gas-sorption experiments often result in ‘open’ systems in which the adsorbate gas is either flowed over the sample at a constant rate or maintained at a constant pressure, with no ability to determine quantitatively the amount adsorbed by the sample or the continuing rate of adsorption. Chevreau *et al.* (2015 ▸) described a more advanced setup involving a computer-controlled manometric dosing system which constantly monitors the pressure inside a sample space of known volume after a known quantity of gas is introduced into the space, allowing the adsorbed quantity to be calculated at all points in time. Powder diffraction data collected during isothermal stepwise dosing with this apparatus allow both equilibrium and non-equilibrium states to be characterized within a broad and controlled range of guest concentrations, yielding detailed information about guest-dependent responses of the framework system.

**Figure 4:**
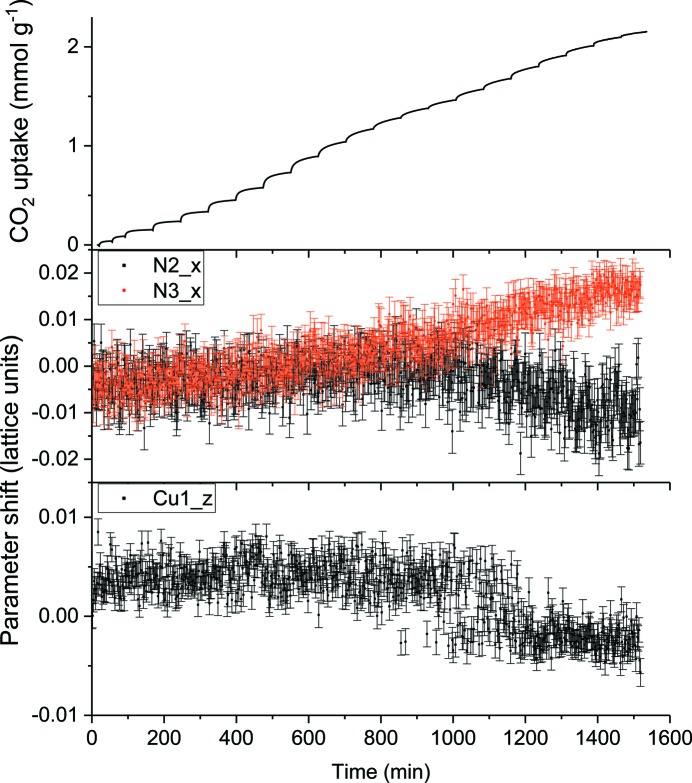
Evolution of selected atomic parameters refined against neutron powder diffraction data collected during the stepwise dosing of Cu_3_(cdm)_4_ with CO_2_. The naturally slow uptake kinetics of the material lead to almost continuous CO_2_ uptake over the later stages of the experiment, with equilibration usually not reached before the next dose is applied (top panel). (New data obtained by some of the present authors.)

The effectiveness of the quantitative approach to *operando* adsorption studies is demonstrated by an analysis carried out in part by some of the present authors of sequential neutron powder diffraction data sets collected for Cu_3_(cdm)_4_ (cdm = carbamoyl­dicyano­methanide) during stepwise adsorption of CO_2_ up to a pressure of 6 bar at 273 K. Diffraction data were recorded every 2 min using the WOMBAT high-intensity neutron powder diffractometer at the OPAL reactor facility in Australia. Due to the slow adsorption kinetics of Cu_3_(cdm)_4_ loaded with more than 0.5 mmol g^−1^ CO_2_, full equilibration was not achieved within the maximum time allowed for any subsequent dosing step (Fig. 4 ▸, top panel), such that the uptake profile over the remainder of the experiment resembled a slow continuous rate of adsorption and only non-equilibrium states were probed.

**Figure 5:**
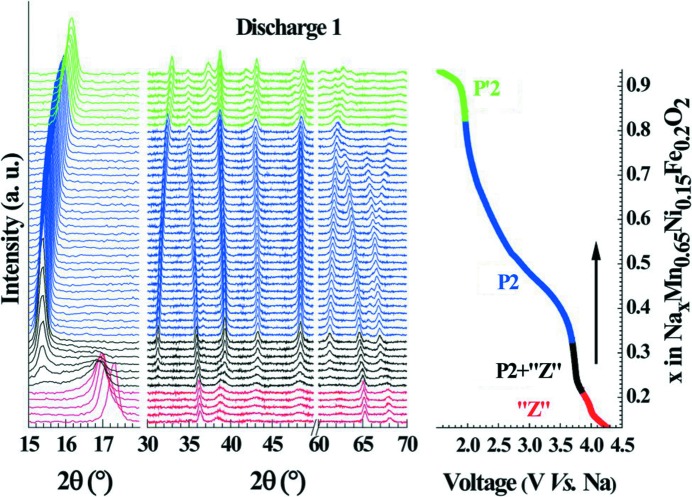
(Left) *Operando* laboratory-based X-ray powder diffraction data recorded during cycling of Na_0.67_[Mn_0.65_Ni_0.15_Fe_0.2_]O_2_ at a rate of *C*/20, along with (right) sodium content *versus* voltage for the first discharge. Colour codes relate to the electrochemical profile on the right. Reprinted with permission from Talaie *et al.* (2015 ▸). Published by the Royal Society of Chemistry.

The data were of sufficient quality to perform full Rietveld refinements of the Cu_3_(cdm)_4_ framework, including independent isotropic atomic displacement parameters and fractional coordinates for each framework atom, and to refine a global occupancy factor for the first of two successive CO_2_ binding sites identified in a previous neutron powder diffraction study of the material equilibrated with known CO_2_ concentrations (McCormick *et al.*, 2014 ▸). Detailed examination of the evolution of some refined parameters revealed a subtle structural transformation occurring as the CO_2_ loading increased from 1.5 to 1.9 mmol CO_2_ g^−1^ (Fig. 5 ▸). Each of these parameters adopts a roughly bimodal distribution of values over the course of the transition. Many of the atoms involved are located near the interaction between Cu^I^ and the cyanide groups, which have been implicated in an anisotropic negative thermal expansion mechanism that is partially suppressed by CO_2_ loading (Chevreau *et al.*, 2015 ▸; J. E. Auckett, personal communication).

Importantly, the fact that the structural parameters oscillate between two states in this range shows the probable co­existence of both states on some length scale, suggestive of local disorder based on different short-range concentrations of CO_2_ in the unsaturated host material. Diffraction experiments of only the equilibrated host–guest system may fail to identify this even when sampling CO_2_ in the 1.5–1.9 mmol g^−1^ range, and thus fail to determine the distribution of states over short time scales. Furthermore, equilibration of the system would be expected to lead to a decrease in local disorder as the CO_2_ guests distribute themselves across the sample, such that the local arrangement would in fact approach the average structure observed. Thus it is clear that less information about the local framework response to CO_2_ under real-time loading conditions could be obtained from an *in situ* diffraction experiment restricted to studying the equilibrium system.

A detailed comparison of data obtained during changing guest concentration with that obtained during static guest concentration and its implications for the study of these systems will be presented in future work, in the context of understanding the real-world functionality of these systems as sorbents.

### 2.5. Summary of advancements: powder diffraction measurements of non-equilibrium framework–guest systems   

Powder diffraction measurements performed on porous framework materials during guest incorporation/release are relatively recent, with experiments prior to about 2010 focusing predominantly on measurements of the equilibrium system as a function of temperature and/or guest concentration at discrete values. More recently, there has been a shift in experimental approach to study guest incorporation in porous framework materials away from the equilibrium system, with reports of real-time measurements beginning to appear in the literature. Reductions in the time scale of such measurements and the improving quality of information contained within each measurement have enabled large advances in insight into the mechanisms by which porous framework materials incorporate and release guest molecules. Such advances have been made possible by the use of high-flux synchrotron X-ray sources and the development of faster detection capabilities on high-intensity neutron powder diffractometers, as well as by the utilization of complementary measurements providing additional information about the system composition, such as those presented in Section 2.3. In the future, efforts should be directed towards performing these complementary measurements simultaneously with powder diffraction measurements. The progression from simple sealed systems or dynamic flow systems with the adsorbate in excess towards more quantitative guest-loading controls, such as those used in Section 2.4, also allows more accurate compositional information to be accessed and greatly enhances the level of structural detail that can subsequently be obtained from such measurements.

## 3. Battery materials   

Since their commercialization in 1991, rechargeable lithium ion batteries have revolutionized the way we live. Despite worldwide research efforts, such batteries remain plagued with problems, including limited lifetime and charge retention, restricting their application in emerging technologies such as electric vehicles, military applications and aerospace industries. Rechargeable batteries function by moving positively charged ions between two electrodes that temporarily host them. When these ions move from one electrode to the other during charge or discharge, the concentration of ions in both electrodes changes over a very large range and the electrode materials must accommodate these changes. Generally, the electrode structure responds to varying ion concentration either by separating into two phases with different ion concentrations or by forming a solid solution where the ion concentration changes continuously across a single phase. These two mechanisms of electrode response directly impact how much charge can be delivered (capacity) and how many times this can be done reversibly (cycle life). Two-phase behaviour is correlated with higher capacity decay and a shorter cycle life compared with solid-solution behaviour, predominantly due to phase boundary movement resulting in particle pulverization.

Typically, the performance of rechargeable batteries is limited by the positive electrode material, the mass of which dictates the battery capacity. A large research effort is aimed at improving the performance of positive electrodes, with strategies of chemical modification, including doping, proving useful. Complex multi-element doping with near neighbours in the periodic table is often involved, and it is not uncommon to have four transition metals at the same site, leading to complex cation ordering and highly complicated structures. Electrode powders often also present as mixtures of two or more phases. Given this complexity, there are several approaches to the structural investigation of electrode function, starting with the detailed structural characterization of the electrode powder and proceeding to how that structure changes as a function of the concentration of charge-carrying ions. These methods include *ex situ* powder diffraction of samples produced through synthetic laboratory methods with varying concentrations of charge-carrying ions, as well as *ex situ* characterization of powders extracted from batteries equilibrated at a particular state of charge (ion concentration).

The use of both *in situ* and *operando* powder diffraction for the structural analysis of battery materials has increased rapidly, with powder diffraction being used to investigate electrode powders *in situ* within a battery that is equilibrated at a particular state of charge, and *operando* non-equilibrium studies targeting the electrode material within a battery during charge and discharge cycling. Measurement of the electrode powder under equilibrium conditions is easier to perform, but may give a misleading picture of electrode function. It is increasingly being demonstrated that the equilibrium electrode system is very different to the non-equilibrium one, as shown by the results of *operando* studies that have led to deep insights into the mechanism of charge transfer within electrodes (Grey & Tarascon, 2017 ▸).

Ideally, real-time powder diffraction data should be collected fast enough to probe the material structure over small changes in ion concentration during electrochemical cycling, such as that occurring at low *C*-rates (the current rate or *C*-rate is defined as the reciprocal of the time taken in hours to charge or discharge a battery completely). However, the need to explore reactions at high *C*-rates imposes a limitation on this. A further, more obvious, challenge to the *operando* powder diffraction analysis of battery materials is that components of the battery other than the material of interest can contribute to the diffraction signal in some way, obscuring the detail that is sought. Both cell geometry and homogeneity are particularly relevant for absorption effects, with angle-dependent variation in X-ray or neutron absorption resulting in incorrect powder diffraction intensities and limiting the crystallographic detail that can be obtained. Such effects commonly present in the background of a powder diffraction pattern. Absorption effects differ significantly between X-ray and neutron powder diffraction, and cell designs that optimize the powder diffraction coming from the component of interest within a cell must be compatible with the type of radiation used as well as with the instrument geometry.

To our knowledge, the first studies of battery materials within electrochemical cells using powder diffraction were reported using X-rays by Gustafsson *et al.* (1992 ▸), paving the way for further work by the same group (Bergström, Gustafsson & Thomas, 1998 ▸) that aimed to improve the signal from the component of interest. A flat-cell design was used in transmission mode to expose only selected components of interest within the lithium polymer battery during *in situ* X-ray diffraction experiments. In the same year, the first *in situ* neutron diffraction experiment of a functioning battery was also reported by this group (Bergstöm, Andersson *et al.*, 1998 ▸). Following these pioneering experiments, dramatic advances in experimental approaches, including cell designs and instrumentation offering better structural detail and temporal resolution, were subsequently developed. Prior to 2016 there were nearly 600 peer-reviewed papers published which discuss the use of *operando* powder diffraction for the mechanistic study of electrodes within functioning batteries. This dramatic increase in the number of battery publications using *operando* and real-time approaches reflects the importance of the understanding of electrode function gained by this method in the development of battery technologies. The use of X-ray powder diffraction (both laboratory and synchrotron) is more common than neutron powder diffraction, undoubtedly as a consequence of the limited availability of neutron facilities and the difficulty of preparing suitable batteries for such experiments. Nevertheless, the elemental contrast offered by neutrons makes them necessary for the structural examination of many battery materials, and they are particularly useful for *operando* experiments where their high penetration allows the examination of whole commercial-sized batteries.

### 3.1. In-house laboratory-based X-ray sources: 20–30 min resolution   

*Operando* battery research using laboratory-based X-ray powder diffraction has seen a renaissance in recent years, with approaches and instrumentation advancing both the time resolution for measurements and the quality of the obtained data to improve our understanding of battery function. The primary restriction of X-ray powder diffraction for the analysis of batteries is beam attenuation. In-house X-ray experiments on batteries are usually limited to low-energy X-rays (8 keV for Cu *K*α), restricting penetration into the cell and resulting in relatively long collection times and poor temporal resolution. Following the first powder diffraction experiments on a flat-geometry polymer battery (Gustafsson *et al.*, 1992 ▸), the experimental setup was improved (Bergström, Gustafsson & Thomas, 1998 ▸) through the addition of an attachment designed specifically for the Stoe & Cie GmbH STADI position-sensitive detector X-ray powder diffractometer, allowing the exposure of selected battery components to the X-ray beam. A dramatic decrease in the acquisition time of reasonable-quality diffraction data for batteries obtained using laboratory X-ray instruments has followed, paving the way for true *operando* investigations on batteries during charge–discharge cycling. For example, *operando* powder diffraction experiments of the Na_0.67_[Mn_0.65_Ni_0.15_Fe_0.2_]O_2_ electrode material within a homemade half-cell mounted on a PANalytical Empyrean diffractometer in Bragg–Brentano geometry using Cu *K*α radiation and a PIXcel detector with an Ni *K*β filter enabled data to be acquired every 30 min during cycling at *C*/20 (a concentration increment of 0.02 Na in the material) (Talaie *et al.*, 2015 ▸). These data allowed the extraction of lattice parameters (Fig. 5 ▸).

Aside from homemade cells, several types of X-ray-transparent electrochemical cells, usually half-cells, have been proposed (as summarized by Sottmann *et al.*, 2016 ▸). One such cell developed for synchrotron experiments (Sottmann *et al.*, 2016 ▸) was used to study LiMn_1.5_Ni_0.5_O_4_ with a laboratory-based Bruker D8 A25 powder diffractometer equipped with an Mo source, focusing mirror optics and a Lynxeye XE high-energy detector, enabling data in the angular range 7–36° to be acquired in 20 min with a *C*/10 rate (a concentration increment of 0.033 Li in the material). No analysis was performed on these data, since it was a proof-of-principle experiment for the cell. Another cell, also developed for synchrotron experiments (Leriche *et al.*, 2010 ▸), was used with a PANalytical diffractometer operating with a Cu *K*α source to study Na_1.86_Fe_3_(PO_4_)_3_ with a sodium counter-electrode at *C*/10 and *C*/20 rates, and although not explicitly reported, data appear to have been acquired for longer than 1 h (Essehli *et al.*, 2016 ▸). Data analysis revealed lattice-parameter changes that indicated the removal of sodium from the tunnels running along the *c* axis.

**Figure 6:**
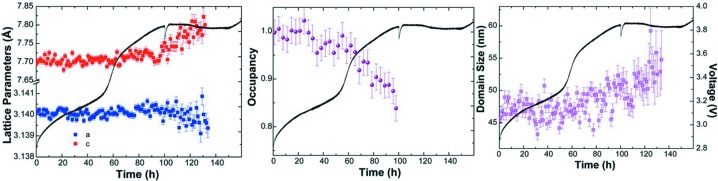
Charging of electrochemically formed Li_2_O_2_, showing the time evolution of lattice parameters, lithium occupancy and average domain size, with the corresponding voltage profile also shown. Reprinted and adapted with permission from Ganapathy *et al.* (2014 ▸). Copyright (2014) American Chemical Society.

A greater level of detail can be obtained from *operando* X-ray powder diffraction analysis of relatively simpler systems, such as the Li–O_2_ battery. Ganapathy *et al.* (2014 ▸) developed a cell for this purpose, and X-ray diffraction measurements were performed on a PANalytical X’Pert Pro PW3040/60 diffractometer with Cu *K*α radiation operating at 45 kV and 40 mA in the angular 2θ range 31−71°, with data recorded in 30 min exposures. Importantly, the data were of sufficient quality to allow the extraction of lattice parameters, lithium occupancy and average domain size for the material, and revealed that the oxidation of electrochemically generated Li_2_O_2_ in the system occurs in two stages, in contrast with the one-step mechanism for bulk crystalline (commercial) Li_2_O_2_ (Fig. 6 ▸). This work was extended recently to understand the electrochemical formation mechanism of Li_2_O_2_ and LiOH (Li *et al.*, 2017 ▸).

### 3.2. Synchrotron X-ray sources: 4–60 s resolution   

Flexible control of the beam energy of synchrotron-based X-ray sources yields dramatically better penetration and faster collection times relative to their laboratory-based counterparts. This provides the opportunity of exploring in greater detail the phase evolution of battery materials, especially at high rates of transformation. The main drawback with synchrotron X-rays, as with laboratory X-ray sources, is that of sample penetration and grain sampling. While many electrochemical cells have been developed to address these issues, the modifications sometimes result in compromised function.

**Figure 7:**
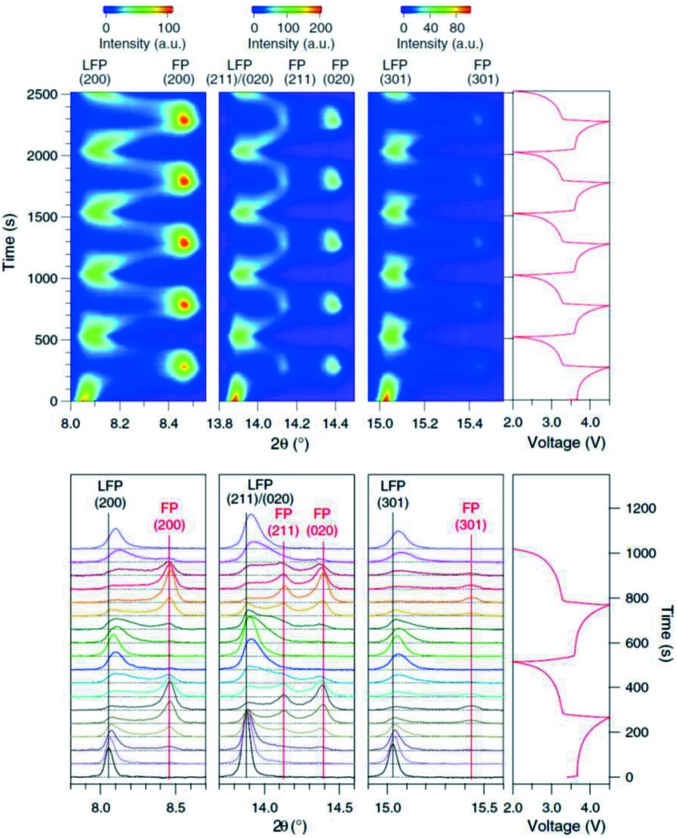
(Top) *Operando* synchrotron powder diffraction data for an LiFePO_4_||Li cell cycled at *C*/10 with data collected every 4 s for the first five cycles and (bottom) the derived phase evolution. LFP is LiFePO_4_ and FePO_4_ is denoted FP. Reprinted with permission from Liu *et al.* (2014 ▸). Copyright (2014) AAAS.

The AMPIX cell, developed specifically for synchrotron X-ray powder diffraction analysis (Borkiewicz *et al.*, 2012 ▸), has been heavily used by battery researchers, commonly at the APS facility in the USA. The AMPIX cell preferentially operates with a free-standing electrode, as a pellet or self-supporting film without a current collector, and with a charge-carrying ion metal as counter-electrode (lithium and sodium have been used). The cell allows data to be collected to *Q* ≃ 19.0 Å^−1^ [*Q* = (4π/λ)sin(θ/2), where θ is the scattering angle and λ is the wavelength of the incident radiation]. An example of research utilizing this popular cell is that reported by Liu *et al.* (2014 ▸) examining further the metastable structures and phase evolution of LiFePO_4_. Performed on the 17-BM beamline of the APS with a high-intensity configuration and fast-read detector, using the AMPIX cell and with Li as the counter-electrode, data were acquired in the 2θ range 1–25° in 4 s, resulting in increments of 0.011 Li at *C*/10. The data were of sufficient quality for whole-powder pattern fitting, revealing the non-equilibrium solid-solution phase Li*_x_*FePO_4_ (0 < *x* < 1) spanning the entire range between LiFePO_4_ and FePO_4_ (Fig. 7 ▸). This work further confirmed that measurements of the equilibrium system are insufficient for the characterization of electrode operational mechanisms.

This work was recently extended to explore structural changes in the NaFePO_4_ system, again using the AMPIX cell (Xiang *et al.*, 2017 ▸). Data were collected every 15 min with a 60 s exposure time at *C*/20 to probe increments of lithium of 0.0125 within the material. Whilst the data were of sufficient quality for Rietveld refinements, the sodium ion occupancy was determined through fitting of Vegard’s law to the unit-cell parameter evolution.

Following from the success of the AMPIX cell, a tubular electrochemical cell for spatially resolved *operando* X-ray scattering and spectroscopic studies, the radially accessible tubular *in situ* X-ray (RATIX) cell, was developed (Liu *et al.*, 2016 ▸). The RATIX cell is compatible with the electrode composition/fabrication and architecture used in standard electrochemical cells, such as electrodes deposited on current collectors, and is an advance over the AMPIX cell.

#### 3.2.1. Simultaneous measurements   

X-ray absorption near-edge spectroscopy (XANES) and extended X-ray absorption fine structure (EXAFS) measurements are often necessary to understand battery material behaviour, and are commonly combined with powder diffraction measurements in battery research. Such measurements allow details of the electronic and local structures of electrode materials to be correlated with phase evolution and average structural changes during redox reactions. Sottmann *et al.* (2016 ▸) developed a method enabling quasi-simultaneous *operando* synchrotron-based powder diffraction, XANES and EXAFS measurements of batteries. The setup is available on the Swiss–Norwegian Beamline BM01B at the European Synchrotron Radiation Facility, France. Typical diffraction profiles are obtained in less than a minute, whereas it takes about 15 min for a high-resolution acquisition in the angular range 5.5–25° with poor counting statistics. A XANES measurement of the Ni *K* edge (8.3 keV) takes about 4 min and an EXAFS measurement takes about 7 min. This approach gives unparalleled insight into the structural function of electrodes and was used in pioneering work to identify a series of structural phase transitions related to electronic changes of the Ni in the ordered LiMn_1.5_Ni_0.5_O_4_ high-potential positive-electrode material for Li-ion batteries (Sottmann *et al.*, 2016 ▸).

**Figure 8:**
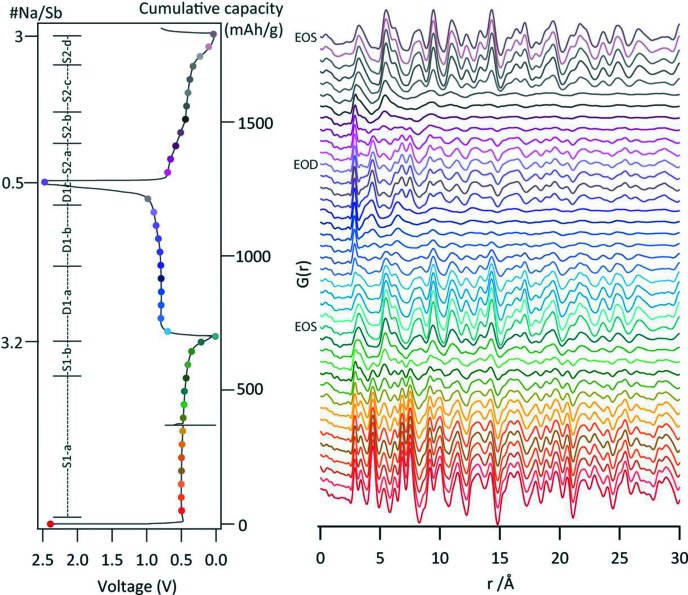
Discharge−charge curves for Sb-containing cells and the corresponding *operando* PDF measurements. Reprinted with permission from Allan *et al.* (2016 ▸). Published by the American Chemical Society.

Nanosizing is adopted to enhance material reactivity through increased surface area and reduced diffusion path lengths and is a particularly important strategy for improving electrode material performance, but results in structures that are difficult to characterize using conventional powder diffraction. Total scattering methods such as pair-distribution function (PDF) analysis are being increasingly applied to characterize electrode materials, and in recent years this has also been successfully implemented using *operando* approaches. Research into electrode local structure using *operando* pair-distribution analysis measurements was first achieved by Hua *et al.* (2014 ▸) using the AMPIX cell. This study examined the conversion reaction of CuF_2_ as pellets with Li metal as the counter-electrode. Total scattering data were collected in transmission geometry on the 11-ID-B beamline at the APS using an amorphous Si-based two-dimensional detector with an X-ray energy of 58 keV. The study reported changes approximately every hour (20 data sets at *C*/20) and revealed the structural detail of asymmetric equatorial and axial Cu—F bonding, reflecting a Jahn–Teller distortion for Cu^2+^, as well as other local bonding information critical for understanding the material’s function. Since this work, the approach has been advanced further to allow its combination with conventional powder diffraction. A study of the lithiation/delithiation of the LiVOPO_4_ nanocomposite using the 11-ID-B beamline at the APS utilized 3 min data acquisitions alternating between PDF and X-ray powder diffraction every 15 min at 0.06 *C* (equivalent to 0.015 Li intervals) (Lin *et al.*, 2016 ▸). Further *operando* PDF measurements on beamline 11-ID-B using this setup were performed to examine the sodiation of antimony as a negative electrode for sodium-ion batteries (Allan *et al.*, 2016 ▸). Cells were cycled at *C*/20 and data were collected every 45 min with an exposure time of 3 min (an increment of 0.066 Na per Sb; Fig. 8 ▸).

### 3.3. Neutron sources: 1 min resolution   

Neutrons are essential for characterizing the structure of many battery materials. Whilst the neutron scattering mechanism enables elemental contrast and penetrating power benefiting the structural characterization of battery components, the incoherent neutron scattering cross section of hydrogen (∼80.3 barn; 1 barn = 100 fm^2^) in the organic electro­lytes and separators typically used in rechargeable batteries produces a high background which masks the useful coherent neutron scattering signal of the targeted electrodes. The traditional step-scan type acquisition of typical neutron powder diffractometers and the order of magnitude lower scattering of neutrons relative to X-rays have hampered the uptake of *operando* neutron powder diffraction for the analysis of battery materials. Although the first *in situ* analysis of materials within batteries essentially constituted equilibrium-state analysis (Bergstöm *et al.*, 1998 ▸; Berg *et al.*, 2001 ▸; Rodriguez *et al.*, 2004 ▸; Rosciano *et al.*, 2008 ▸; Colin *et al.*, 2010 ▸), these works pioneered the method, spurring the next generation of truly *operando* neutron powder diffraction investigations of batteries.

Much work has been done to overcome the difficulties of achieving *operando* neutron powder diffraction of batteries through cell designs and the use of deuterated electrolytes and hydrogen-poor separators. The development of neutron powder diffractometers with improved flux and faster detection capabilities over larger solid angles has occurred in parallel with this effort, and a variety of electrochemical cells designed for various geometries of neutron instrumentation have been developed. In particular, WOMBAT has been used extensively for *operando* battery research, being arguably the fastest reactor-based neutron powder diffractometer and featuring an area detector covering 120° in 2θ. For instruments such as WOMBAT, annular cells with neutron-transparent walls such as the vanadium can roll-over type (Sharma *et al.*, 2011 ▸; Brant *et al.*, 2014 ▸) and approaches using layered pouch-type batteries that reduce variations in neutron path lengths through the cell to the detector (Pang & Peterson, 2015 ▸) have been developed. Such cells also accommodate the relatively large amounts of electrode materials that are mandated by the use of neutrons to compensate for the reduced scattering power of neutrons relative to X-rays. The penetration gained by neutrons allows full cells to be examined, including the very common commercial 18650 type that typically contains ∼20 g of active electrode material. This allows sampling of the bulk electrode and therefore probes the operational mechanism under real-world conditions, while allowing details of the phase evolution of both electrodes to be obtained simultaneously.

#### 3.3.1. Phase evolution   

The first truly *operando* neutron powder diffraction experiment of an electrode within a battery was reported for an unmodified prismatic commercial LiCoO_2_||graphite cell (Sharma *et al.*, 2010 ▸). Data were collected on WOMBAT every 5 min, and whilst a complex phase evolution was revealed, the data quality limited the insights to lattice response and phase composition. Subsequent work on WOMBAT examined the mechanism of lithiation/delithiation of LiFePO_4_, a hotly debated topic (Sharma *et al.*, 2012 ▸). This work collected *operando* neutron powder diffraction on a 5 min time scale to examine LiFePO_4_ housed within a roll-over design battery with lithium as the counter-electrode inside a vanadium cell (essentially a null-matrix material for neutron diffraction) and with deuterated electrolyte (see the summary of cell designs given by Sharma *et al.*, 2015 ▸). The data provided the first direct experimental evidence for competitive solid-solution and two-phase reactions occurring in this material. Importantly, this occurrence was theoretically predicted to occur under non-equilibrium conditions, and the measurement could therefore only be performed in *operando*.

Realisation of the full potential of crystal structure analysis using *operando* neutron powder diffraction, as obtained from Rietveld refinement, has been hampered by low data quality as a result of the incoherent scattering from hydrogen, as well as absorption effects arising from cell inhomogeneity and geometries incompatible with the neutron diffractometer. Bianchini *et al.* (2013 ▸) noted that, for neutron powder diffraction data from any of the electrochemical cells developed for battery components, ‘it is almost impossible to perform a good quality multi-phase pattern refinement’, and they consequently developed a cell made of Ti/Zr (a null-matrix material for neutron diffraction) for use on the high-flux diffractometer D20 at the ILL in France. The cell was used to examine the behaviour of Li_1.1_Mn_1.9_O_4_ and contained deuterated electrolyte with Li as the counter-electrode. Data could be recorded down to 10 min, but the desired Rietveld-quality data required 1 h.

Ongoing research efforts have continued to improve the quality of *operando* neutron powder diffraction data obtained for batteries. A neutron pouch cell was developed for WOMBAT that mimicked an annular cell in that it provided equal neutron scattering path lengths through the cell to the curved area detector (Pang & Peterson, 2015 ▸). This cell was used to study the non-commercial LiNi_0.5_Mn_1.5_O_4_||Li_4_Ti_5_O_12_ electrode combination (Pang, Sharma *et al.*, 2014 ▸) and features deuterated electrolyte with a stacked layer arrangement of electrodes that enables a larger volume of electrode to be probed, allowing high-quality data to be acquired in 5 min. The phase and lattice-parameter evolution of both electrodes were easily derived from these data. In particular, the dis­ordered LiNi_0.5_Mn_1.5_O_4_ was found to exhibit solid-solution behaviour during the Ni^2+^/Ni^3+^ redox transition and a two-phase reaction between Li*_x_*Ni_0.5_Mn_1.5_O_4_ and Ni_0.25_Mn_0.75_O_2_ products during the Ni^3+^/Ni^4+^ redox transition. This insight explained the electrochemical performance of the electrode. For the other electrode, Li_4_Ti_5_O_12_, which exhibits a purely solid-solution reaction, the details of the Ti–O octahedra are of fundamental importance to its electrochemical function. Whilst the oxygen positional parameter could not be refined, clear intensity changes in the data supported the structural operational mechanism that lithium ions at tetrahedral (8*a*) sites transfer to octahedral (16*c*) sites while O atoms move away from the transition metals as the average valence of Ti decreases from 4^+^ to 3.4^+^ during charge.

The pouch cell for WOMBAT (Pang & Peterson, 2015 ▸) was also used to examine the Li_1+*x*_ *M*O_2_ (*M* = Li, Ni, Mn, Fe) composite electrode (Pang, Kalluri *et al.*, 2014 ▸), with a carbon counter-electrode and deuterated electrolyte, again enabling data to be collected in 5 min. The Li_1+*x*_ *M*O_2_ electrode was composed of two phases, and the structural evolution of the complex cation-doped main (Li_0.8_Ni_0.2_)(Li_0.2_Ni_0.13_Mn_0.33_Fe_0.33_)O_2_ phase with space group 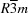 was found to exhibit a solid-solution reaction during the Ni^2+^/Ni^3+^/Ni^4+^ redox transitions, which is mechanistically different from isostructural electrodes. These data supported the refinement of the oxygen positional parameter in this main phase, revealing that increasing octahedral distortion enabled the material to deform structurally, explaining its structural stability.

The preparation of specialist cells for neutron diffraction experiments is complicated and commercial cells are used wherever possible. Importantly, the larger amount of electrode in many commercial cells mitigates the difficulties of obtaining a good signal from the electrode of interest due to the hydrogen-rich electrolyte and separator they contain. Unfortunately, using commercial batteries also limits the battery chemistry being explored to those already available, but nevertheless excellent information concerning the phase and lattice-parameter evolution of electrodes has been obtained.

Fully commercial large-format LiFePO_4_||C pouch batteries were studied on the High-Resolution Fourier Diffractometer at the Frank Laboratory of Neutron Physics of the Joint Institute for Nuclear Research in Russia (Bobrikov *et al.*, 2014 ▸). This instrument is housed at the IBR-2 reactor with one of the most intense neutron fluxes available and data could be acquired every 2.5 min, from which the phases and phase transitions of the electrodes were successfully identified, in particular for the carbon electrode.

The phase evolution of electrodes within 18650-type Li_*x*_(Ni_0.5_Mn_0.3_Co_0.2_)O_2_||C batteries was explored using the high-resolution powder diffractometer (SPODI) at the Heinz Maier-Leibnitz (FRM II) research reactor in Germany (Dolotko *et al.*, 2014 ▸). Although it took 30 min to acquire meaningful data on this high-resolution instrument, the approach was taken to collect these shorter data sets continuously in combination with longer 4 h acquisitions at fixed battery states of charge. In this way, a more detailed structural picture of electrode function was gained, at the expense of broad operational insight.

**Figure 9:**
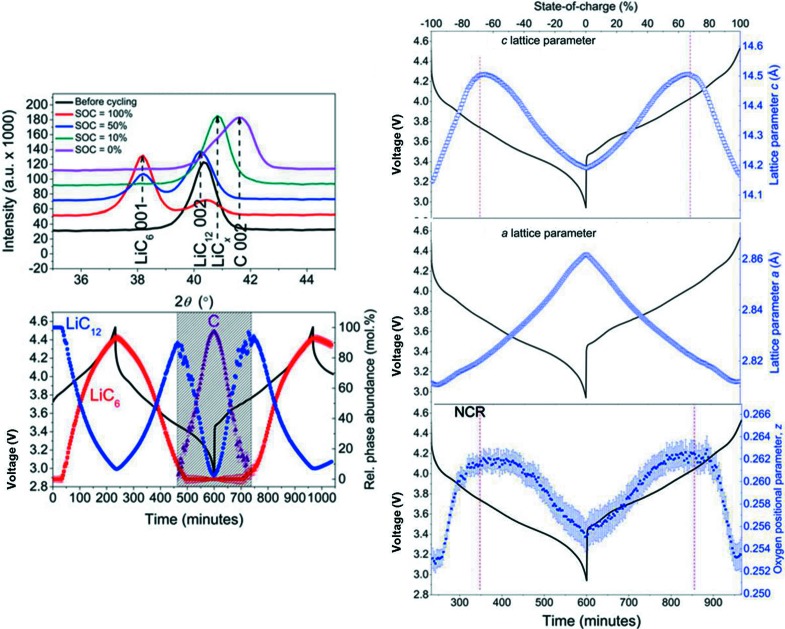
Phase evolution of the (left) carbon and (right) Li(Ni,Co,Al)O_2_ electrodes within an 18650-type commercial battery obtained using *operando* neutron powder diffraction on WOMBAT. Also shown are the associated voltage profile and extracted oxygen positional parameter of the Li(Ni,Co,Al)O_2_ structure. Adapted from Pang *et al.* (2015 ▸).

18650-type Li(Ni,Co,Al)O_2_||C batteries were explored on WOMBAT and data collected in 3 min. The combination of the large amount of electrode and the annular geometry ideally suited to WOMBAT meant that more detail of the phase evolution of the electrodes could be gained than in previous experiments, particularly for the carbon electrode. Notably, this work revealed that an intermediate LiC*_x_* phase could be observed as formed through a solid-solution like two-phase reaction between LiC_12_ and C during lithiation (Pang *et al.*, 2015 ▸) (Fig. 9 ▸). Whilst full unconstrained Rietveld refinement of the Li(Ni,Co,Al)O_2_ structure was not supported by the data, the details of the oxygen bond length were obtained by refinement of the oxygen positional parameter, and the evolution of the *a* lattice parameter was explained by the decreased attraction between the lower oxidation-state transition metal (Ni) and O ions, causing transition metal oxygen octahedra expansion.

Narrower annular commercial 10440-type batteries containing a three-phase mixture of Li(Ni,Mn,Co)O_2_, LiCoO_2_ and LiMn_2_O_4_ with a carbon counter-electrode were also investigated using *operando* neutron powder diffraction on the multidetector diffractometer at the Paul Scherrer Institute in Switzerland (Nazer *et al.*, 2016 ▸). Data were collected for 3–5 min and enabled the details of phase and lattice evolution to be derived. Similarly, it was also recently demonstrated on WOMBAT that the even larger commercial 26650-type batteries could also be studied successfully using neutron powder diffraction, with data acquired in 1 min (Goonetilleke *et al.*, 2017 ▸).

At present, it is relatively straightforward to obtain information regarding the phase and lattice evolution of battery components using *operando* neutron powder diffraction on a minute time scale, using a range of commercial and specialized neutron cells. Such experiments have recently been expanded to include other parameters such as the temperature and current dependence of these evolutions (Sharma *et al.*, 2017 ▸).

#### 3.3.2. Structural detail   

**Figure 10:**
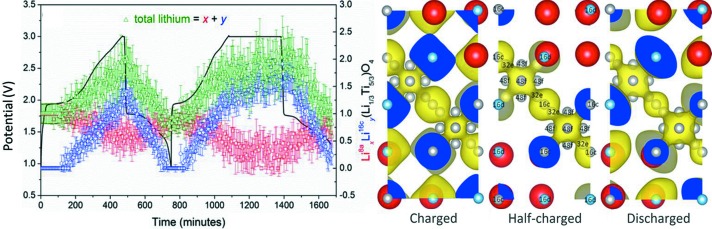
(Left) Li site occupancy at the 8*a* and 16*c* sites for Li_4_Ti_5_O_12_ during cycling. (Right) The nuclear density arising from Li (yellow; O is red and Ti is blue) at particular crystallographic sites (labelled, grey) within the structure, shown for the material within the battery at the charged, half-charged and discharged states. Adapted from Pang, Peterson *et al.* (2014 ▸).

As researchers have gained experience with the *operando* neutron powder diffraction analysis of battery materials, the quality of data obtained while still maintaining an *operando* approach has increased. Perhaps of most importance to understanding electrode function is the determination of charge carrier concentration and location within the electrode material, which was first achieved for lithium by Sharma *et al.* (2013 ▸). This work used an unmodified pouch-type commercial LiCoO_2_||C battery, mounted on WOMBAT in a way similar to that later described by Pang & Peterson (2015 ▸), providing roughly equal path lengths for neutrons through the cell to all parts of the detector. Data were collected in 5 min and were of sufficient quality to support full Rietveld analysis, revealing the lithium location and concentration during charge/discharge. Further work using the custom-designed pouch cell on WOMBAT (Pang, Peterson *et al.*, 2014 ▸) investigated the well known near zero-strain Li_4_Ti_5_O_12_ material coupled with LiFePO_4_. Data collected in 5 min revealed the site-dependent Li concentration and the oxidation state of redox-active Ti obtained from the Ti—O bond length in the Li_4_Ti_5_O_12_ material, in addition to the phase evolution and lattice-parameter changes in both electrodes. Nuclear density arising from Li revealed the Li diffusion pathway in Li_4_Ti_5_O_12_. In addition to the 16*c* and 8*a* crystallographic sites, the 32*e* site was implicated in the Li diffusion pathway, bridging between the 16*c* and 8*a* sites, lowering the barrier energy as predicted theoretically (Fig. 10 ▸).

Some neutron diffractometers, such as the Stress-Spec instrument at FRM-II in Germany, can define a volume within the sample from which scattering is detected and isolate scattering from a particular battery component. This approach can result in improved data quality, at the expense of greater breadth of information. Experiments using an 18650-type (LiNi_1/3_Mn_1/3_Co_1/3_O_2_)||C battery on this instrument collected data over the reduced angular range 30–40° in 2θ in 5 min acquisitions (Zinth *et al.*, 2014 ▸). In this way information targeting the lithiated carbon phases was gained, yielding the lithium concentration in carbon.

The null-matrix cell of Bianchini *et al.* (2013 ▸) for the D20 instrument at the ILL was used to study the spinel electrode Li_1+*x*_Mn_2–*x*_O_4_ for *x* = 0, *x* = 0.05 and *x* = 0.10 (Bianchini *et al.*, 2014 ▸). *Operando* data collected over 30 min enabled the Li site occupancy and O position to be determined, as well as the phase evolution. This study revealed that the lithiation mechanism was highly dependent on *x*. A similar level of detail was also obtained for the lithiation of the high-voltage LiNi_0.4_Mn_1.6_O_4_ material using this cell, with data again acquired for 30 min but this time using the high-resolution instrument D2B at the ILL (Bianchini *et al.*, 2015 ▸).

**Figure 11:**
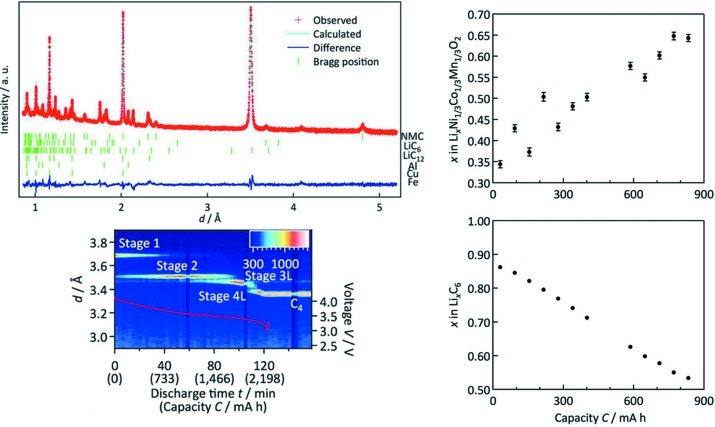
(Left) The high-quality *operando* neutron powder diffraction data obtained on the SPICA diffractometer, and (right) the results derived from Rietveld refinements detailing lithium content in both electrodes. Adapted from Taminato *et al.*, 2016 ▸.

The latest instrument to be used for *operando* structural analysis of battery materials is the special-environment neutron powder diffractometer, SPICA, at the Materials and Life Science (MLF)/Japan Proton Accelerator Research Complex (J-PARC) facility in Japan. The time-of-flight method provides significant advantages for the *operando* analysis of battery materials, offering higher flux that translates into higher temporal resolution (limited to the pulse length) as well as improved angular resolution. On SPICA, every neutron event is recorded and the temporal resolution chosen after the experiment, with data enabling full Rietveld refinements gained on the minute time scale. In addition to phase transition information, as revealed for 18650-type LiNi_1/3_Co_1/3_Mn_1/3_O_2_||C cells (Shiotani *et al.*, 2016 ▸), detailed information for the 18650-type Li(Ni,Mn,Co)O_2_||C cell (Taminato *et al.*, 2016 ▸) was gained on SPICA at least as quickly as on other neutron diffractometers but with unprecedented spatial resolution, enabling lithium location and content to be determined (Fig. 11 ▸). Data collected at various *C*-rates (0.05, 0.1, 0.5, 1 and 2 *C*) indicated the presence of inhomogeneous reactions along several directions within the material at higher currents, as well as a relaxation process occurring during a high current drain discharge (Taminato *et al.*, 2016 ▸).

### 3.4. Summary of advancements: powder diffraction measurements of non-equilibrium battery systems   

Time-resolved powder diffraction is a well established approach for understanding battery materials function, and measurements performed during charge–discharge cycling of battery materials housed within electrochemical cells have been described in nearly 600 peer-reviewed papers prior to 2016. Both synchrotron and neutron radiation are commonly applied for this research, with the necessary trade-off between time and spatial resolution using traditionally slower neutrons being somewhat mitigated by modern high-intensity instruments such as WOMBAT at the OPAL facility in Australia and, most recently, SPICA at the MLF/J-PARC facility in Japan.

Advances in the level of structural detail that can be gained from these experiments have been substantial, predominantly as a result of specialist electrochemical cell designs. Importantly, each cell must be optimized for a particular instrument, so a substantial range of standard cells has arisen with construction themes common to the type of radiation (X-ray or neutron) and the instrument optics.

Finally, any additional detail that can be obtained simultaneously with the powder diffraction measurement of battery materials will deepen the understanding of the mechanism by which guest species are incorporated or released by the system. An example of this is provided by the experimental setup on the Swiss–Norwegian Beamline BM01B at the European Synchrotron Radiation Facility in France, which is designed to allow simultaneous powder diffraction and X-ray absorption measurements of battery electrodes during charge–discharge cycling.

## 4. Outlook   

Time-resolved techniques are essential to understanding non-equilibrium processes in energy materials, with powder diffraction becoming an increasingly used tool with which to explore energy-critical guest–host systems such as porous sorbents and battery electrodes. These time-resolved powder diffraction studies probe guest transport mechanisms directly in the non-equilibrium guest–host system, avoiding the misleading or incomplete results often obtained from measurements of the system at equilibrium. Increases in the speed of instrumentation have reduced the time resolution for measurements of the changing non-equilibrium system to the sub-minute timeframe.

Whilst real-time powder diffraction is an established tool for the analysis of battery components during charge–discharge battery cycling, the same methods for porous materials used in gas separation and storage are in the early stages of application. The distribution of states that are present in non-equilibrium systems can complicate the structural analysis of powder diffraction data, especially when the exact composition of the (changing) system is unknown. Whilst the number of intercalated charge-balancing ions can be inferred from the simultaneously measured electrochemical response of a battery, real-time quantification of gas uptake in porous samples presents greater challenges. The further development of equipment facilitating fully quantitative guest dosing will enable observed changes in the host–guest system to be related more easily to concentration-dependent host–guest and guest–guest interactions. The availability of high-speed instrumentation and the construction of sample environments capable of supporting multiple controlled parameters and simultaneous *in situ* measurement types are also greatly advantageous to this research. In particular, it is hoped that future developments will eventually also lead to *operando* characterization experiments performed during uptake of gas mixtures by porous materials, as such experiments replicate the conditions of real-world gas separation processes more closely and lead to a better understanding of the kinetic features of sorbent selectivity.
